# Supplementary material for: Application of a Physiologically Based Pharmacokinetic Model to Develop a Veterinary Amorphous Enrofloxacin Solid Dispersion
Source: Pharmaceutics. 2021 Apr 22;13(5):602. doi: 10.3390/pharmaceutics13050602 (PMC8143505; doi:10.3390/pharmaceutics13050602)
Supplement: Supplementary file 1 [file pharmaceutics-13-00602-s001.zip › pharmaceutics-1174313-supplementary.pdf]

pharmaceutics

MDPI

Supplementary Materials: Application of a Physiologically Based Pharmacokinetic Model to Develop a Veterinary Amorphous Enrofloxacin Solid Dispersion

Kaixiang Zhou, Meixia Huo, Wenjin Ma, Kun Mi, Xiangyue Xu, Samah Attia Algharib, Shuyu Xie and Lingli Huang

Table S1. Physiological parameters used in the PBPK model for enrofloxacin in swine.

Parameter	Abbreviation	Model value	Published value
Body weight (kg)	BW	20.0	92.5 ± 4.0[c]
Cardiac output (L/h/kg)	QCC	5.0	8.70 ± 1.62[c]
**Tissue volume**[a]			
Small intestine	VSiC	0.036	0.0126 ± 0.0023[c]
Arterial blood	VartC	0.0156	0.0156[d]
Venous blood	VvenC	0.0444	0.0444[d]
Liver	VLC	0.0247	0.0201 ± 0.0025[c]
Kidney	VKC	0.004	0.0037 ± 0.001[c]
Muscle	VMC	0.4	0.426 ± 0.039[c]
Fat	VFC	0.32	0.32[d]
Lung	VLuC	0.01	0.0077 ± 0.0014[c]
Rest of body	VrestC	0.1813	0.1813[d]
**Blood flow**[b]			
Liver	QLC	0.2725	0.243 ± 0.075[c]
Kidney	QKC	0.12	0.114 ± 3.2[c]
Muscle	QMC	0.251	0.342 ± 0.346[c]
Fat	QFC	0.1275	0.1275[d]
Rest of body	QrestC	0.229	0.229[d]

Note: [a]fraction of body weight, unitless; [b]fraction of cardiac output, unitless. [c]Lin et al, 2020 (the experimental measured data); [d]Lin et al, 2016.

Table S2. Chemical-specific parameters used in the PBPK model for enrofloxacin in swine.

Parameter	Abbreviation	Model value	Published value[a]
**Absorption rate constant (/h)**			
Gastric emptying	Kst	2.0	1.0
Intestinal absorption	Ka	0.55	0.55
Intramuscular	Kim	0.0	0.5
Subcutaneous	Ksc	0.0	0.1
**Tissue: plasma partition coefficient (unitless)**			
Liver	PL	4.3	4.3
Kidney	PK	5.5	5.5
Muscle	PM	3.0	3.0
Fat	PF	0.53	0.53
Lung	Plu	4.3	4.3
Hepatic metabolic rate [(/h*kg)]	KmC	0.045	0.045
Plasma protein binding rate	PB	0.46	0.46
Fecal elimination rate constant (/h)	Kfeces	0.01	0.01
Urinary elimination rate constant (L/h/kg)	KurineC	0.12	0.12

Note: [a]Lin et al, 2016.

Enrofloxacin PBPK model code in AcslX format

```
PROGRAM


INITIAL

! code that is executed once at the beginning of a simulation run goes here


!! Physiological parameters

! Blood flow rates (fraction of cardiac output)

CONSTANT QCC = 5 ! Cardiac output index (L/h/kg), also blood flow of lung, from Upton (2008)

CONSTANT QLC = 0.2725 ! liver, average from Buur et al. (2005) and Upton (2008)

CONSTANT QKC = 0.12 ! kidney, average from Buur et al. (2005) and Upton (2008)

CONSTANT QMC = 0.251 ! Muscle, average from Buur et al. (2005) and Upton (2008)

CONSTANT QFC = 0.1275 ! Fat, average from Buur et al. (2005) and Upton (2008)


! Tissue volumes (fraction of body weight)

CONSTANT BW = 55 ! Kg, body weight was study-specific; The actual value in present study

CONSTANT VLC = 0.0247 ! liver, average from Buur et al. (2005) and Upton (2008)

CONSTANT VKC = 0.004 ! Kidneys, average from Buur et al. (2005) and Upton (2008)

CONSTANT VMC = 0.40 ! Muscle, average from Buur et al. (2005) and Upton (2008)

CONSTANT VFC = 0.32 ! Fat, adipose tissue, average from Buur et al. (2005) and Upton (2008)

CONSTANT VLuC = 0.01 ! Lungs, average from Buur et al. (2005) and Upton (2008)

CONSTANT VBloodC = 0.06 ! Blood, average from Buur et al. (2005) and Upton (2008)

CONSTANT VSiC = 0.036 ! Small intestine, from Lautz et al. (2020).


!! Mass transfer parameters (Chemical-specific parameters)

! Chemical molecular weights and unit conversion factors, from PubChem

CONSTANT MW = 359.4 ! g/mol, enrofloxacin

CONSTANT MWmol = 2.78 ! µmol/mg, enrofloxacin, from mg to µmol

CONSTANT MWmg = 0.36 ! mg/µmol, enrofloxacin, from µmol to mg


! Kinetic constants
```

```
! Oral absorption and fecal elimination rate constants for enrofloxacin
CONSTANT Kst = 2 ! /h, gastric emptying rate constant
CONSTANT Ka = 0.55 ! /h, intestinal absorption rate constant
CONSTANT Kfeces = 0.01 ! /h, intestinal transit rate constant (fecal elimination rate constant)

! IV infusion/injection rate constants
CONSTANT Timeiv = 0.01 ! h, IV infusion/injection time

! IM absorption rate constants (set parameter value equal to 0.0 when not used in a particular simulation)
CONSTANT Kim = 0.0 ! /h, intramuscular absorption rate constant

! SC absorption rate constants
CONSTANT Ksc = 0.0 ! /h, subcutenous absorption rate constant

! Partition coefficients for enrofloxacin (PC, unitless)
! The values from Buur et al. 2005 were used as initial values for further estimation.
CONSTANT PL = 4.3 ! Liver:plasma PC
CONSTANT PK = 5.5 ! Kidney:plasma PC
CONSTANT PM = 3 ! Muscle:plasma PC
CONSTANT PF = 0.53 ! Fat:plasma PC
CONSTANT PLu = 4.3 ! Lung:plasma PC
CONSTANT Prest = 8 ! Rest-of-body:plasma PC

! Partition coefficients for the ciprofloxacin (usually designated as the marker residue) (PC, unitless)
CONSTANT PL1 = 4.3 ! Liver:plasma PC
CONSTANT PK1 = 5.5 ! Kidney:plasma PC
CONSTANT PM1 = 4.3 ! Muscle:plasma PC
CONSTANT PF1 = 0.53 ! Fat:plasma PC
CONSTANT PLu1 = 4.3 ! Lung:plasma PC
CONSTANT Prest1 = 8 ! Rest-of-body:plasma PC
```

```
! Percentage plasma protein binding (unitless), Buur et al. (2005)
CONSTANT PB = 0.46 ! Percentage of enrofloxacin bound to plasma proteins

! Metabolic rate constants
CONSTANT KmC = 0.045 ! /(h*kg), liver metabolic rate constant of the enrofloxacin

! Urinary elimination rate constants
CONSTANT KurineC = 0.12 ! L/h/kg, for enrofloxacin
CONSTANT PDOSEoral = 5 ! mg/kg
CONSTANT PDOSEiv = 0 ! mg/kg
CONSTANT PDOSEim = 0 ! mg/kg
CONSTANT PDOSEsc = 0 ! mg/kg

END ! INITIAL
DYNAMIC

ALGORITHM IALG = 2
NSTEPS NSTP = 10
MAXTERVAL MAXT = 1.0e9
MINTERVAL MINT = 1.0e-9
CINTERVAL CINT = 0.1

DERIVATIVE
! code for calulating the derivative goes here
! Cardiac output and blood flows to tissues (L/h)
QC=QCC*BW ! Cardiac output
QL=QLC*QC ! Blood flow to the liver
QK=QKC*QC ! Blood flow to the kidney
QM=QMC*QC ! Blood flow to the muscle
QF=QFC*QC ! Blood flow to the fat
Qrest = QC-QL-QK-QM-QF ! Blood flow to the rest of body
```

```
! Tissue volumes (L)
VL=VLC*BW ! Liver
VK=VKC*BW ! Kidney
VM=VMC*BW ! Muscle
VF=VFC*BW ! Fat
VLu=VLuC*BW ! Lung
VBlood=VBloodC*BW ! Blood
Vven=VBlood*0.74 ! Venous blood
Vart=VBlood*0.26 ! Arterial blood
Vrest = BW-VL-VK-VM-VF-VLu-VBlood ! Rest of body
VSi=VSiC*BW ! Small intestine

! Dosing amounts (mg converted to µmol)
DOSEoral=PDOSEoral*BW*MWmol ! µmol
DOSEiv=PDOSEiv*BW*MWmol ! µmol
DOSEim=PDOSEim*BW*MWmol ! µmol
DOSEsc=PDOSEsc*BW*MWmol ! µmol

! Multiple oral dosing using the PULSE/EXPOSURE function
CONSTANT tlen = 0.001 ! Length of exposure, oral, iv, im, or sc (h/day)
CONSTANT tinterval = 12 ! administration interval, varied dependent on the exposure paradigm (h)
CONSTANT Dstart = 0.0 ! Initiation day of exposure (day)
CONSTANT Dstop = 5 ! Termination day of exposure (day)
CONSTANT MAXT = 1.0 ! maximum comm. interval
CONSTANT CINTC = 0.1 ! Communication interval
CINT = CINTC ! Communication interval
Tsim=TSTOP*24 ! Tstop in hours
DS=Dstart*24 ! Initiation time point of exposure (h)
Doff=(Dstop-Dstart)*24 ! Exposure duration (h)
TimeOn=Dstart*24 ! Initiation time point of exposure (h)
```

```
TimeOff=Dstop*24+tlen ! Terminatison time point of exposure (h)
Exposure=PULSE(0,tinterval,tlen)*PULSE(DS,Tsim,Doff) ! Exposure paradigm
RDOSEoral=(DOSEoral/tlen)*Exposure ! Administrataion rate
RAST=RDOSEoral-Kst*AST ! Rate in the stomach
AST=Integ(RAST,0)!0.0 or Doseoral if the initial dose is twice as the subsequent dose.
RAI=Kst*AST-Ka*AI-Kfeces*AI ! Rate in the intestine
Rfeces=Kfeces*AI ! Fecal elimination rate
Afeces=Integ(Rfeces,0.0) ! Amount eliminated throµgh feces
AI=Integ(RAI,0.0) ! Amount in the intestine
CAI=AI/VSi ! Concentration of the enrofloxacin in small intestine, µmol/L
CAImg=AI*MWmg ! Concentration of the total enrofloxacin in small intestine, unit conversion from µmol/L to mg/L (µg/g)
RAO=Ka*AI ! Oral absorption rate
AAO=Integ(RAO,0.0) ! Amount absorbed

! Single IV dosing to the venous
IVR=DOSEiv/timeiv
RIV=IVR*(1.0-step(timeiv)) ! Intravenous injection rate
AIV=Integ(RIV,0.0) ! Amount injected

! Single IM exposure
Rim=Kim*Aimsite ! Intramuscular absorption rate
Aim=Integ(Rim,0.0) ! Amount absorbed via IM route
Rimsite=-Kim*Aimsite ! Rate of changes in the amount of the drug in the injection site
Aimsite=Integ(Rimsite,Doseim) ! Amount of the drug remained in the injection site

! Multiple IM exposure (if needed)
!RDOSEim=(DOSEim/tlen)*Exposure
!Rimsite=RDOSEim-Kim*Aimsite
!Aimsite=Integ(Rimsite,0.0)
!Rim=Kim*Aimsite
!Aim=Integ(Rim,0.0)
```

```
! Single SC exposure
Rsc=Ksc*Ascsite ! Subcutaneous absorption rate
Asc=Integ(Rsc,0.0) ! Amount absorbed via SC route
Rscsite=-Ksc*Ascsite ! Rate of changes in the amount of the drug in the injection site
Ascsite=Integ(Rscsite,Dosesc) ! Amount of the drug remained in the injection site

! Metabolic rate
Km=KmC*BW ! h-1
! Urinary elimination rates
Kurine=KurineC*BW ! L/h, for the enrofloxacin
Kurine1=Kurine1C*BW ! L/h, for the ciprofloxacin

! Venous blood/plasma
RV=QL*CVL+QK*CVK+QM*CVM+QF*CVF+Qrest*CVrest+Riv+Rim+Rsc-QC*CV ! Rate, µmol/h
AV=Integ(RV,0.0) ! Amount, µmol
CV=AV/Vven ! Concentration of the total enrofloxacin (free plus bound), µmol/L
CVfree=CV*(1-PB) ! Concentration of the enrofloxacin that is free, µmol/L
CVbound=CV*PB ! Concentration of the enrofloxacin that is bound, µmol/L
CVmg=CV*MWmg ! Concentration of the total enrofloxacin (free plus bound), unit conversion from µmol/L to mg/L (µg/g)

! Arterial blood/plasma
RA=QC*CVLu-QC*CAfree ! Rate, µmol/h
AA=Integ(RA,0.0) ! Amount, µmol
CA=AA/Vart ! Concentration of the total enrofloxacin (free plus bound), µmol/L
CAfree=CA*(1-PB) ! Concentration of the enrofloxacin that is free, µmol/L
CAbound=CA*PB ! Concentration of the enrofloxacin that is bound, µmol/L
ABlood=AV+AA ! Amount of the total drug in the blood, µmol

! Lung compartment
RALu=QC*(CV-CVLu) ! Rate, µmol/h
```

ALu=Integ(RALu,0.0) ! Amount, µmol

CLu=ALu/VLu ! Concentration of the total enrofloxacin in the lung, µmol/L

CVLu=CLu/PLu ! Concentration of the total enrofloxacin in venous blood drained from the lung, µmol/L

! Liver compartment

RL=QL*(CAfree-CVL)+RAO-Rmet ! Rate, µmol/h

AL=Integ(RL,0.0) ! Amount, µmol

CL=AL/VL ! Concentration of the total enrofloxacin in the liver, µmol/L

CVL=CL/PL ! Concentration of the total enrofloxacin in the venous blood drained from the liver, µmol/L

CLmg=CL*MWmg ! Concentration of the total enrofloxacin in the liver, mg/L (µg/g)

! Metabolism of the parent compound in the liver compartment

Rmet=Km*CL*VL ! Total hepatic metabolic rate, µmol/h

Rmet1=Rmet*Frac ! Hepatic metabolic rate to the ciprofloxacin, µmol/h

Rmet2=Rmet*(1-Frac) ! Hepatic metabolic rate to other minor metabolites, µmol/h

Amet=Integ(Rmet,0.0) ! Amount of the enrofloxacin that is metabolized in the liver, µmol

Amet1=Integ(Rmet1,0.0) ! Amount of the ciprofloxacin that is produced in the liver, µmol

Amet2=Integ(Rmet2,0.0) ! Amount of other minor metabolites that are produced in the liver, µmol

! Kidney compartment

RK=QK*(CAfree-CVK)-Rurine ! Rate, µmol/h

AK=Integ(RK,0.0) ! Amount, µmol

CK=AK/VK ! Concentration of the total enrofloxacin in the kidney, µmol/L

CVK=CK/PK ! Concentration of the total enrofloxacin in the venous blood drained from the kidney, µmol/L

Ckmg=Ck*MWmg ! Concentration of the total enrofloxacin in the kidney, mg/L (µg/g)

! Urinary excretion of the parent compound

Rurine=Kurine*CVK ! Rate, µmol/h

Aurine=Integ(Rurine,0.0) ! Amount, µmol

! Muscle compartment

```
RM=QM*(CAfree-CVM) ! Rate, µmol/h
AM=Integ(RM,0.0) ! Amount, µmol
CM=AM/VM ! Concentration of the total enrofloxacin in the muscle, µmol/L
CVM=CM/PM ! Concentration of the total enrofloxacin in the venous blood drained from the muscle, µmol/L
CMmg=CM*MWmg ! Concentration of the total enrofloxacin in the muscle, mg/L (µg/g)

! Fat compartment
RF=QF*(CAfree-CVF) ! Rate, µmol/h
AF=Integ(RF,0.0) ! Amount, µmol
CF=AF/VF ! Concentration of the total enrofloxacin in the fat, µmol/L
CVF=CF/PF ! Concentration of the total enrofloxacin in the venous blood drained from the fat, µmol/L
CFmg=CF*MWmg ! Concentration of the total enrofloxacin in the fat, mg/L (µg/g)

! Rest-of-body compartment
Rrest=Qrest*(CAfree-CVrest) ! Rate, µmol/h
Arest=Integ(Rrest,0.0) ! Amount, µmol
Crest=Arest/Vrest ! Concentration of the total enrofloxacin in the rest-of-body, µmol/L
CVrest=Crest/Prest ! Concentration of the total enrofloxacin in the venous blood drained from the rest-of-body, µmol/L

! Mass balance for the enrofloxacin
Qbal=QC-QL-QK-QM-QF-Qrest ! Blood flow balance
Tmass=ABlood+AL+AK+AM+AF+Arest+ALu+Aurine+Amet ! Total amount in the body, µmol
Bal=AAO+AIV+AIM+ASC-Tmass ! Mass balance, input minus output should be equal to zero at all time

END ! DERIVATIVE
! Add discrete events here as needed
! DISCRETE
! END

! code that is executed once at each communication interval goes here
CONSTANT TSTOP = 230
```

```
TERMT (T .GE. TSTOP, 'checked on communication interval: REACHED TSTOP')

END ! DYNAMIC
TERMINAL
! code that is executed once at the end of a simulation run goes here
END ! TERMINAL
END ! PROGRAM
```
